# Hæmorrhoids and Their Treatment

**Published:** 1881-08

**Authors:** A. Chargé

**Affiliations:** Paris, France


### HÆMORRHOIDS AND THEIR TREATMENT.

DR. A. CHARGÉ, PARIS, FRANCE.

(Continued from page 195.)

ANACARDIUM.—Hæmorrhoids seem to cause frequent and urgent desire for a stool, but the desire passes away with the effort, without an evacuation. Rectum feels as if plugged up. [*Kali. bich.* and *Lach.*, have sensation of plug in anus; cannot sit.] Deficient power of rectum; even soft stools cause straining to pass them. [*Alum.*, *Calc.*, *China.*, *Carbo. veg.*, *Sil.*, *Sepia*, *Psor.*, *Phos. ac.*] Moisture from anus. [*Baryta c.*, *Carbo. an.*, *Carbo. v.* (acrid), *Caust.*, *Coloc.*, *Natr. m.*, *Sepia.*] Itching at anus; stools of pale color (Hg.); profuse hemorrhage when at stool. *Pain about umbilicus as of a dull plug squeezed into intestines.* (Hg.)

Concomitant symptoms: Nervous prostration; weakness of intellect, especially for proper names, in mornings; feebleness of sight; weak digestion, with no appreciable gastric disease; hypochondriac, with gastric disorders; erections during day, at night seminal emissions without lascivious dreams. Females are nervous and hysterical.

Ant. Crud.—Hæmorrhoids that have been bleeding for some time, or a white mucoid discharge, which leaves a yellow stain, also irritates surrounding parts, and causes pricking and burning. This discharge is more profuse at night than during the day. When there is constipation, or when the stool is preceded by colic, there is some blood mixed with the mucous discharge.

*Concomitant Symptoms:* Predominance of gastric disorders; fœtid eructation; bitter or tasting of ingesta; nausea and sometimes vomiting; fœtid gas; small appetite; constipation alternating with diarrhœa [especially in the aged]; tongue coated white, a milky white; this coating differs from that of *Kali. bich.* and *Pulsat.*, though not any thicker; gastric troubles and concomitant cutaneous affections; frequent congestion of blood to head; stiffness of back, and rheumatic pains in various parts of the body.

Apis Mell.—Especially indicated when the pains are marked by a continued *stinging* sensation. [*Carbo. an.* and *Caust.* have piles burning and stinging, worse walking; *Natr. m.* has stinging pain; *Sulph. ac.* burning, stinging, tearing pain. But these drugs are more or less aggravation from cold and amelioration by heat, which is vs. *Apis.*] Excessive smarting around anus; excoriation [throbbing in rectum, with sensation as if stuffed full, *Æsculus hipp.*]; frequent desire to go to stool; frequent stools, watery, of dark blood [greenish-yellow, olive-green, with bright *red* lumps; thin yellow, with weakness; stool with every motion of body, as if anus were constantly open. *Phos.* Hg.]; urine dark or bloody; dysuria. *Apis* has aggravation of the pains from heat and amelioration from cold and cold water; nocturnal aggravation.

Arsenicum.—Burning pains; sensation of burning is so strong that patient compares it to a burning coal;* worse at night. The hæmorrhoidal tumors are swollen, inflamed, prolapsed and bunched; bleed from the least touch. The hæmorrhoids are irreducible, even to the most skillful manipulation, and are even strangulated; rhagades often appear at various points. Their color is violet [*Phos.*, *Verat.*] or blackish [*Lach.*, *Rhus.*]. The discharge is ichorous; fissures with burning pain. [Anus red and sore; parts around excoriated by the evacuations. Hg.]. Tongue dry, cracked [tongue red, *edges red*, with imprints of teeth. Hg.]. Violent thirst, patient

* Pain as if caused by a burn; Ant. crud., Ars., *Carbo veg.*, Caust., Creos., Cyclam., Euphorb., Hyos., Rhus., Sec.-cor., Stram.—Bœnninghausen.

drinking little and often. Pain in lumbar region, as if back would break; impossible to stoop. Heat; trembling; with great weakness and prostration of all the vital forces. The patient feels constantly a sensation of heat in the veins and skin. Excess of alcoholic drink is an occasional cause of Arsenic hæmorrhoids. [The Arsenic hæmorrhoids have a stitching burning when walking or sitting, but *not* at stool; worse at night; wakes patient; relieved by heat.]

BELLADONNA.—Hæmorrhoids prolapsed; irreducible on account of a spasmodic contraction of the sphincter ani. The tumors are of a bright red color, and bleed profusely, worse from the lightest touch. The patient constantly seeks various (grotesque) positions to lessen the pain caused by compression. Unbearable pains in the loins, and a sensation in the sacrum as if the bone were broken. Urinary troubles; micturition frequent and difficult; burning in bladder and urethra. Pulse full; face red, purplish. Congestive headache.

BORAX.—Hæmorrhoids discharging slimy mucus, mixed, of yellowish or brownish color. Itching and pricking at anus and in rectum. Indicated in children with liquid stools, green like chopped spinach.

BRYONIA.—In bilious persons and in the hot summer weather, "is indicated more certainly when the disease causes a sensation of fullness in eighth lumbar region, and when this fullness changes during movement to a sharp, cutting pain, eased by rest, and seems to be caused by congestion of spleen."—Hartman. Aching piles: Stool hard and black; dry, as if burnt; scanty; straining to pass it.

CALCAREA CARB.—Hæmorrhoids prolapsed, much congested; painful while walking, eased by sitting; they render defecation very painful. Profuse hemorrhage is as prominent for *Calcerea* as for *Belladonna.* Fœtid sweat around anus; prickling in rectum, as if from ants [as of ascarides.—Jahr]. Heat in rectum and at anus.

Especially indicated if there exist such a sympathy between the hæmorrhoids and the head, that the latter is disturbed when the hæmorrhoidal flow ceases; this trouble of the head is generally vertigo, felt especially when going up-stairs, with dullness and heaviness of head. In women with menses early and profuse. Is an excellent remedy for the bad effects of suppressed hæmorrhoidal flow (Nux.), when these symptoms are present: continued vertigo, with fear of falling; tottering gait; pressing and stupefying headache, heaviness of head; weakness of memory. *Frequent* stools, first hard, then soft, and lastly, liquid. Profuse sweat of the feet, which is fœtid, and excoriates the skin.

CAPSICUM AN.—Burning and itching at anus; cutting and smarting during defecation, even when the stool is liquid. The hæmorrhoids are very painful, and consequently very much inflamed, with free discharge of blood, and after the blood a discharge of mucus. Hæmorrhoids blind or flowing, with burning pain; piles, swollen, itching and throbbing; stools of dysenteric character; burning in abdomen. Urinary troubles, such as tenesmus, frequent and futile urging to urinate; smarting and burning in the urethra during and after urination. Those who constantly use *Capsicum* as a condiment suffer from hæmorrhoids, with burning at anus and constriction of rectum. Suppressed hæmorrhoidal flow, causing melancholy. [Hg.]

By so doing they would very soon perceive that *Capsicum* possesses no privileges not enjoyed by our other drugs. These accidental discoveries often occur thus: one day (why do not these days of *light* come more frequently?) some one mentions a drug that possesses a great efficacy in curing painful and inflamed hæmorrhoidal tumors; this drug is *Capsicum annum.* For a wonder the announcement is believed and a commission appointed to investigate; this commission experiments and makes its report. The report is favorable. I quote it *verbatim:* "We observed that after the second day of using this drug there was a very appreciable amelioration of the symptoms; and the disease was ordinarily cured in a few days. After administering it for some days the pains were eased, the size of the tumors lessened, and very soon they dried up and healed." When it is asserted that *Capsicum* cures hæmorrhoids, the Academy willingly bears witness to it. Experiments on healthy men.

CARBO VEG.—In old external hæmorrhoids, tumor large and of bluish color. Incessant sweat; a mucous discharge, giving out a very bad odor. Tardy stools, accompanied by violent burning pains, and sometimes by a discharge of blood. [Acrid corrosive moisture discharged from rectum, while urinating; a great amount of hot, moist and offensive flatus, ameliorated by its escape (*China, Lyco., nux, Puls., verat.*); soft stool, passed with difficulty (*Alum, China, Silicea, Sepia, Phos-ae.*); stools burning, light colored, covered by filamentous yellow mucus, last part bloody; stool in fragments, tough and scanty, with urging and tingling in rectum; putrid smelling stools.—(*Ars., China, Puls., Silicea, Sulph.*)]

*General Symptoms:* Rush of blood to head; lancinating pains in kidneys; worse from a false step; stiffness in lumbar region; cutting burning pains under the scapulæ, in fore-arms, and sometimes in the knees. Loss of appetite, fullness in stomach, bad taste in mouth and bad odor. Intolerable flatulence. Gout. Adynamic conditions, with no fever.

Causticum.—Hæmorrhoids impeding walking on account of the pain they cause; also impede stool; are swollen, itch; stitching in them; burn and sting, worse walking and when thinking of them; Stool shines, is first hard, then soft; passed better when standing. Tumors sensitive to touch. Frequent and ineffectual urging to stool with pain, anxiety and redness of face. Painful pustule near anus, with discharge of pus and blood. Fissures which render walking very painful.

